# Durable Leukemic Remission and Autologous Marrow Recovery with Random Chromosomal Abnormalities after Allogeneic Hematopoietic Stem Cell Transplantation for Chronic Lymphocytic Leukemia

**DOI:** 10.1155/2019/9710790

**Published:** 2019-01-03

**Authors:** Hidekazu Nishikii, Naoki Kurita, Atsushi Shinagawa, Tatsuhiro Sakamoto, Manabu Kusakabe, Yasuhisa Yokoyama, Takayasu Kato, Mamiko Sakata-Yanagimoto, Naoshi Obara, Yuichi Hasegawa, Naoya Nakamura, Shigeru Chiba

**Affiliations:** ^1^Department of Hematology, Faculty of Medicine, University of Tsukuba, Tsukuba, Japan; ^2^Department of Hemato-Oncology, Hitachi General Hospital, Hitachi, Japan; ^3^Department of Pathology, Tokai University, Tokyo, Japan

## Abstract

A 38-year-old woman with aggressive clinical course of chronic lymphocytic leukemia (CLL) was treated with 8 courses of R-CHOP. Clinical remission was achieved, while B-cell clonality remained. Allogeneic hematopoietic stem cell transplantation was performed with reduced intensity conditioning (fludarabine and 2-Gy total body irradiation). However, autologous hematopoietic recovery occurred within a month after the transplant. Nevertheless, B-cell clonality became undetectable at 14 days after transplant, which has been kept so for over 10 years with clinical remission. Cytogenetic analyses were repeatedly performed and demonstrated nonclonal chromosomal aberrations, although the patient did not develop any secondary malignancies. One possible explanation for the clinical course is a very short-term allogeneic immune reaction helping eradication of residual CLL cells.

## 1. Introduction

Chronic lymphocytic leukemia (CLL) is a clonal B-cell neoplasm, which is generally slowly progressive but difficult to be cured even with current chemoimmunotherapy. Allogeneic hematopoietic stem cell transplantation (allo-HSCT) is a possible therapeutic choice for disease cure, although the indication of allo-HSCT for CLL is limited because of the advances of treatment options with lower morbidity. Here, we report a case of durable molecular remission of CLL with autologous marrow recovery after allo-HSCT. The patient demonstrated the recovery of recipient-derived hematopoietic system within one month after allo-HSCT but did not show any signs of CLL relapse even 10 years after allo-HSCT. This is a report of a rare case implying that a cure of CLL could be achieved possibly by a very short-term allogeneic immune reaction. Repeated cytogenetic studies after autologous recovery are accompanied with a detailed description of random chromosomal abnormalities.

## 2. Case Presentation

**Figure 1:**
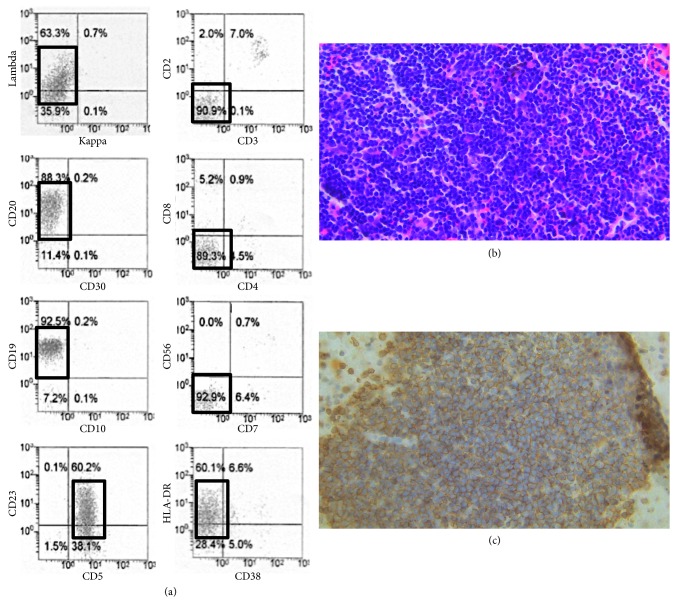
(a). Flow cytometric analysis of peripheral blood at diagnosis. □ means leukemic cell gate. Leukemic cells are positive in IgG*λ*, CD20, CD19, CD23, CD5, and HLA-DR. (b) Hematoxylin and eosin staining of leukemic cells in the bone marrow clot (× 400). (c) Immunostaining of CD20 (× 400).

**Figure 2:**
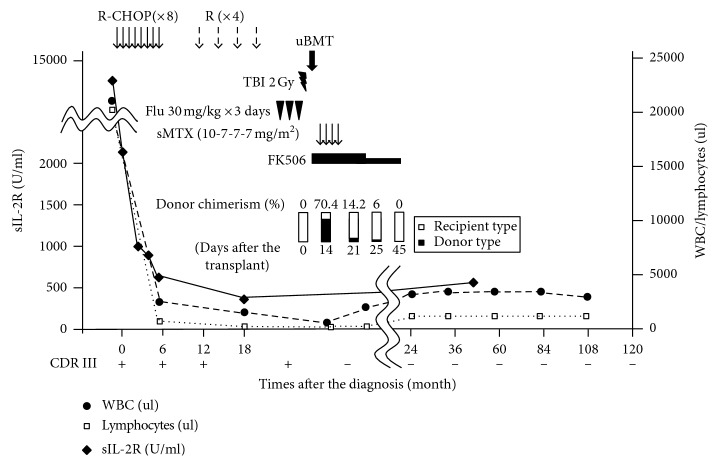
Clinical course. Absolute number or white blood cells (WBC, •), lymphocytes (□), and soluble IL2-receptor (sIL-2R, ◆) were shown in graph. R-CHOP, rituximab, cyclophosphamide, doxorubicin, vincristine, and prednisolone; Flu, fludarabine; TBI, total body irradiation; uBMT, unrelated bone marrow transplantation; sMTX, short-term methotrexate; CDR III, complementarity determining region III.

A 38-year-old woman with systemic lymphadenopathy and fever was admitted to a local hospital. Performance status was 2 according to the Eastern Cooperative Oncology Group criteria [1]. White blood cell count (WBC) was 21 × 10^9^/L, with 91% of nucleated cells being small and mature lymphocytes. Mild anemia (hemoglobin, 10.8 g/dl) and moderate thrombocytopenia (platelet number, 46 × 10^9^/L) were also observed (Table 1). Flow cytometric analysis revealed that the increased lymphocytes were positive for CD5, CD19, CD20, CD23, and IgG*λ* (Figure 1(a)). *IgH/cyclinD1* translocation was negative in fluorescence in situ hybridization analysis. Cytogenetic analysis of bone marrow cells demonstrated the normal karyotype. She also demonstrated elevated levels of aspartate transaminase (156 IU/l; normal 13–30 U/l), alanine transaminase (139 IU/l; normal, 7–23 U/l), lactate dehydrogenase (595 IU/l; normal, 124–232 U/l), and total bilirubin (1.8 g/dl; normal, 0.4–1.5 mg/dl). Soluble interleukin-2 receptor was also markedly elevated (10,400 U/ml; normal, 190–650 U/ml; Table 1 and Figure 2). Contrast computed tomography revealed multiple lymphadenopathy, hepatosplenomegaly, bilateral pleural effusion, and massive ascites. Bone marrow biopsy demonstrated a remarkable infiltration of small mature lymphocytes (57% of the total bone marrow cells). Immunophenotype of the major population was the same as that of peripheral blood lymphocytes. A small number of relatively large, blastic lymphocytes were diffusely observed in the bone marrow. Polymerase chain reaction (PCR) analysis demonstrated a single complementarity determining region (CDR-III) signal, indicating clonal B-cell proliferation. Taken together, she was diagnosed to have CLL (Rai stage IV and Binet stage C).

Because of her aggressive disease presentation and suspect of Richter syndrome, R-CHOP therapy (rituximab 375 mg/m^2^, cyclophosphamide 750 mg/m^2^, daunorubicin 50 mg/m^2^ for one day, and prednisolone 100 mg/body for 5 days) was chosen as an initial therapy. After the treatment, her lymphadenopathy, hepatosplenomegaly, pleural effusion, and ascites were improved and 8 courses of R-CHOP were given. Morphologic CLL cells disappeared in peripheral blood. However, flow cytometric analysis revealed that 0.17% of the total bone marrow cells were leukemic cells and that the same single CDR-III signal as the one detected at the diagnosis was confirmed by PCR. These observations suggested the presence of minimum residual diseases (MRD), even after additional three courses of rituximab monotherapy.

Because of young age, suspect of Richter syndrome, and remaining MRD, the patient was referred to the University of Tsukuba Hospital 21 months after the diagnosis for allo-HSCT. The graft was bone marrow from an unrelated female donor with one-allele mismatched HLA. Nonmyeloablative conditioning regimen was chosen containing fludarabine 30 mg/m^2^ for 3 days and 2-Gy total body irradiation. Tacrolimus and short-term methotrexate were used for graft-versus-host disease (GVHD) prophylaxis (Figure 2). On day 14, neutrophil engraftment was achieved, and the MRD judged by PCR for CDR-III became undetectable with the bone marrow cells. Donor-cell chimerism in the bone marrow was 70.4%, when evaluated by short tandem repeat- (STR-) PCR analysis. However, a progressive neutropenia was observed from day 20, when peripheral blood cells showed decreased donor chimerism down to 14.2%. On day 24, STR-PCR of bone marrow cells revealed 100% recipient type, suggesting rejection of the donor graft. Favorably, however, the multilineage cytopenia was gradually improved, and single CDR-III signal was never detected after the transplant. On day 62, WBC was 34 × 10^9^/L with 42.7% neutrophils and 41.8% lymphocytes. Hemoglobin was 10.2 g/dl, and the platelet number was 146 × 10^9^/L. These observations suggested that autologous bone marrow recover with remission of CLL. No apparent acute GVHD was observed. Leukemic cells were never detected in the bone marrow or peripheral blood in flow cytometry even 10 years after the transplant. There were no chronic GVHD-like symptoms or signs. Cytogenetic studies were repeated 15 times over 10 years for the screening purpose to detect emergence of clonal cells. In total, 299 metaphase cells were analyzed, and abnormalities were found in 50 cells (16.7%, Table 2). Abnormalities in 7q22 were found in four cells at 3 independent studies performed at 53 through 61 months after the transplant. However, the 7q22 abnormalities were detected only temporarily and were never found thereafter until 136 months after the transplant. All the other abnormalities were seen only one time. All this agrees with the concept of random chromosomal abnormalities (RCA) seen in automarrow recovery after receiving high-dose ionizing radiation exposure [2]. Despite the careful follow-up, the patient has been evaluated not to develop any clonal disorders such as myelodysplastic syndromes (MDS). Her blood cell counts and lymphocytes number were within the normal range at the last visit (140 months after the transplant), and there was no dysplastic changes in peripheral blood and bone marrow cells.

## 3. Discussion

CLL, the most common mature B-cell neoplasm in Caucasians, is generally prevalent in elderly people, although it is also present in younger adults. In the current therapeutic strategy for CLL [3–5], the indication of allo-HSCT is very limited. In the report from Fred Hutchinson Cancer Research Center, allo-HSCT with fludarabine plus low-dose total body irradiation regimen improved overall survival (OS; 5-year OS, 50%) and progression-free survival (PFS; 5-year PFS, 39%) in CLL patients [6]. However, the other literatures reported that the risk of graft rejection in nonmyeloablative allo-HSCT for CLL was relatively high (12–25% with T-cell depleted grafts and 5–6% with unmanipulated grafts) [7]. High incidences of infectious complication are also concerned in allo-HSCT for CLL. Moreover, the recent advances of chemoimmunotherapy with lower morbidity decrease the indication of allo-HSCT for CLL. Therefore, allo-HSCT is currently considered only for the relapsing patients with *TP53* mutations and/or del (17p), or patients who are refractory to repeated chemoimmunotherapies [4]. Transformation into more aggressive lymphoma, called as Richter syndrome, also could be considered for the indication of allo-HSCT [8].

In the current case, non-myeloablative allo-HSCT was performed after achieving first hematological remission of CLL based on the aggressive clinical presentation at diagnosis with the suspect of Richter syndrome and the persistence of MRD. Later, Richter syndrome was thought to be inaccurate because the frequencies of blastic lymphocytes at the diagnosis did not fulfill the criteria, and thus, arguments may remain regarding the clinical decision for allo-HSCT. Such patient would be treated with the other treatment options if current chemoimmunotherapies would be applicable. Anyhow, autologous bone marrow recovery resulted. No donor-type hematopoiesis was detectable at the level with STR-PCR, and there was no apparent GVHD. Autologous marrow recovery after allo-HSCT is sometimes observed in patients with chronic myelogenous leukemia, in the majority of those who eventually demonstrate the disease relapse [9, 10]. However, the long-term consequence after the post-allo-HSCT autologous marrow recovery in CLL is unclear. Given that we were not able to find any report describing a long-term outcome after autologous marrow recovery in CLL, the clinical course of the current case is considered to be extremely rare. There is no good evidence-based scenario that explains the mechanism how CLL cells were eradicated and have been controlled for a long time. One possibility may be that a very short-term allogeneic immune reaction soon after the transplantation has helped eradicate residual CLL cells. In any case, low tumor burden and non-myeloablative regimen with very weak cytotoxicity may have caused the recovery of patient's own hematopoietic system and durable remission of disease.

We also showed the results of repeated cytogenetic analyses, demonstrating RCA over 10 years after autologous marrow recovery (Table 2). RCA represents nonclonal chromosomal aberrations and are caused by external factors such as radiation, chemical, or others [11, 12], among which experiences of high-dose ionizing radiation are the most abundant [13, 14]. In the literature regarding cord blood transplant for a lethally irradiated nuclear accident victim [15], clonal and nonclonal chromosomal aberrations within 100 days after the transplant were documented. Given the fact that irradiation exposure is associated with an increased risk for MDS development even 40–60 years after the exposure [2], and careful follow-up is mandatory.

## Figures and Tables

**Table 1:** Laboratory findings at the initial diagnosis.

Peripheral blood	
WBC	**21 × 109/l**
Seg	**3%**
Band	**1%**
Lymph	**70%**
A-lym	**21%**
Mono	**2%**
Eos	0%
Baso	1%
RBC	**308 × 104/*μ*l**
Hb	**10.8 g/dl**
Plt	**46 × 109/l**
*Coagulation*	
PT	12.1 sec
APTT	27.1 sec
Fib	241 mg/dl
AT	86%
FDP	5.1 *μ*g/ml
*Blood chemistry*	
TP	**6.0** **g/dl**
Alb	3.8 g/dl
AST	**156** **U/l**
ALT	**139** **U/l**
LDH	**595** **IU/l**
GGT	**319** **U/l**
ALP	**2022** **U/l**
ChE	**163** **U/l**
T-bil	**1.8** **mg/dl**
D-bil	**1.1** **mg/dl**
AMY	58 U/l
CK	29 U/l
BUN	16 mg/dl
Cre	0.7 mg/dl
Na	142 mEq/l
K	4.0 mEq/l
Ca	9.1 mg/dl
CRP	**1.19** **mg/dl**
*β*2MG	**5.41** **mg/l**
Ferritin	102.5 ng·ml
sIL-2R	**10400** **U/ml**
*Immunology*	
IgG	615 mg/dl
IgA	**50** **mg/dl**
IgM	**17** **mg/dl**
C3	**124** **mg/dl**
C4	36 mg/dl
CH50	**51.5** **U/ml**

Bold means the abnormal laboratory value.

**Table 2:** Random chromosomal abnormalities after autologous hematopoietic recovery.

Days after the transplant (month)	Karyotype (number of cells)	Number of abnormal karyotypes	Number of the metaphase
At diagnosis	46,XX[20]	(0)	(20)
45 (1)	46,XX,t(8;11)(p23;p21)[1] / 46,XX,t(8;22)(p21;p13)[1] / 46,XX[18]	2	20
172 (24)	46,XX,t(2;3)(p23;q29)[1] / 46,XX[19]	1	20
886 (29)	46,XX,add(1)(p11),add(2)(p11.2),−8,add(12)(q13),+mar1[3] / 46,XX [17]	3	20
1257 (41)	46,XX,t(3;3)(p11;p25)[2] / 46,X,t(X;8)(p22.3;q13)[1] / 46,XX,t(11;15)(q23;q15)[1] / 46,XX[15]	4	19
1623 (53)	46,XX,del(7)(q?)[3]^*∗*^ / 46,XX,t(12;15)(q24.1;q22)[1] / 46,XX,add(4)(q12),add(6)(p21),der(6)t(4;6)(q12;q21)[1] / 46,XX[15]	5	20
1819 (59)	46,XX,add(7)(q22)[1]^*∗*^/46,XX,t(1;3)(p36.3;p21)[1] / 46,X,?t(X;16)(q22;q24),t(1;13)(p35.3;q14)[1] / 46,XX,t(13;20)(q14;q13.1)[1] / 46,X,?inv(X)(p11.2q21),t(5;16)(q31;p13.3)[1] / 46,XX[15]	5	20
1861 (61)	46,XX,add(7)(q22)[2]^*∗*^/ 46,XX,del(7)(q22),add(19)(q13.3)[1]^*∗*^/ 46,XX[17]	3	20
2022 (66)	46,XX,t(5;11)(q13;q21)[1] / 46,XX,t(5;13)(q13;q21)[1] / 46,XX,t(5;9)(q22;q34)[1] / 46XX,t(1;15;13;13)(q32;q15;p12;q12),?t(4;11)(p16;q23)[1] / 46, XX[16]	4	20
2169 (71)	46,XX,t(3;3)(p25; q21)[1]/46,XX,t(7;11)(p13;q22)[1] / 46,XX,t(9;16)(q22;q24)[1] / 46,XX,t(16;21)(q12-13;q22)[1] / 46,XX[16]	4	20
2358 (77)	46,XX,t(4;5)(q35;q11.2)[2] / 46,XX,t(3;6)(q24;p25)[1] / 46,XX, add(10)(p13),−15,+mar[1] / 46,XX[16]	4	20
2547 (83)	46,XX,t(2;16)(q13;q24)[1] / 46,XX, t(2;4)(q37;q31)[1] / 46,XX,add(1)(p13),?add(3)(p25),der(22)t(1;22)(p22;q11.2)[1] / 46,XX[17]	3	20
2792 (91)	46,XX,der(1)inv(1)(p13;q21)t(1;5)(p13;q11.2),der(5)t(1;5)(p13;q11.2)[1] / 46,XX[19]	3	20
3184 (104)	46,XX,−4,add(5)(p15),−10,der(11)(q23),−12,−13,+4mar[1] / 46,XX,t(16;22)(q24;q11.2)[1] / 46,XX[18]	2	20
3821 (125)	45,XX,−2,t(3;9)(p25;q22)[1] / 46,XX,inv(11)(p15;q13)[1]/46, XX t(1;5)(p32;q13)[1] / 46,XX,−4,−5,−8,−10,−22,+5mar[1] / 47,XX,+5[1] / 46,XX[15]	5	20
4211 (138)	44,XX,t(1;6)(q12;q21),−4,−13[1] / 46,XX,inv(10)(q11.2q24)[1] / 46,XX[18]	2	20
	Total number of analyzed cells after the transplant	50	299

^*∗*^Potential clone. The results of chromosomal analysis of bone marrow cells at several time points after allo-HSCT are shown above.
